# Storage stability of commonly used haematological parameters at 33 °C: Electronic supplementary material available online for this article.

**DOI:** 10.11613/BM.2018.020901

**Published:** 2018-04-15

**Authors:** Ashish Jain, Sanchit Jain, Neha Singh, Priyanka Aswal, Shweta Pal, Sushant Kumar Meinia, Nilotpal Chowdhury

**Affiliations:** 1 Department of Transfusion Medicine, All India Institute of Medical Sciences, Rishikesh, Uttarakhand, India; 2 M.B.B.S. student, All India Institute of Medical Sciences, Rishikesh, Uttarakhand, India; 3 Department of Pathology and Laboratory Medicine, All India Institute of Medical Sciences, Rishikesh, Uttarakhand, India

**Keywords:** pre-analytical error, erythrocyte indices, laboratory errors, platelet count, sample stability

## Abstract

**Introduction:**

This study aimed to investigate the analytical bias in haematological parameters induced by storage at 33 ºC.

**Materials and methods:**

Blood from the diversion pouch of 20 blood donors were collected in K__2__EDTA vials and stored at 33 ºC. Readings from each vial were taken at 0, 4, 6, 12, 24, 48 and 72 hours after collection on the Sysmex XP-100 analyser (Sysmex Corporation, Kobe, Japan). The percent difference from the baseline readings were calculated and subjected to a Wilcoxon signed rank test at a Holm corrected significance level of 0.05. A median percent difference, which was statistically significant and greater than the maximum acceptable bias (taken from studies of biological variation), was taken as evidence of unacceptable shift. If the median shift was lesser than the maximum acceptable bias, two one-sided Wilcoxon signed rank tests for equivalence were used to determine whether the percent differences were significantly lesser than the maximum acceptable bias.

**Results:**

Haemoglobin, red blood cell count, white blood cell count, mean corpuscular haemoglobin and lymphocyte count showed acceptable bias after storage for at least 24 hours at 33 ºC. Haematocrit, mean corpuscular volume, mean corpuscular haemoglobin concentration, platelet count and mean platelet volume showed unacceptable shift in less than 4 hours when stored at 33 ºC.

**Conclusions:**

Since many haematological parameters show unacceptable bias within 4 hours of sample storage at 33 ºC, the recommended limit of time from collection to processing should be revised for areas where high environmental temperatures are common.

## Introduction

Knowledge of the effects of temperature on blood sample stability is important in a haematology laboratory and blood bank setting. Exposure to high environmental temperatures (*e.g.* above 30 °C), which are quite common in under-resourced settings in tropical countries, may lead to variation and errors in the reporting of different haematology parameters. Guidelines have stated that processing of samples should be done within 6 to 8 hours (*1*, *2*). However, there is scant evidence that samples maintain their analytical stability when stored for such time at high environmental temperatures. Most studies have shown that haemoglobin (Hb), red blood cell count (RBC), white blood cell count (WBC), platelet count (Plt) and mean corpuscular haemoglobin (MCH) are stable for at least 24 hours at 4 °C and between 20 °C and 25 °C (*3*). Mean corpuscular volume (MCV), mean corpuscular haemoglobin concentration (MCHC), red cell distribution width (RDW) and haematocrit (Hct) were stable for at least 6 hours at 4 °C and between 20 °C and 25 °C. However, we found only one study in English on analytical stability providing numerical data for most haematological parameters on samples stored above 30 °C (*4*).

Therefore, the present study estimated the analytical stability of different haematological parameters over a period of 72 hours in samples stored at 33 °C.

## Materials and methods

### Subjects

This study was carried out in the Department of Transfusion Medicine and Blood Bank after obtaining ethical clearance from the institutional ethics committee. In this study, samples were collected from the diversion pouches of 20 apparently healthy blood donors not using anti-platelet drugs and meeting criteria set by regulatory agencies (*5*). There was no contamination with blood bag anticoagulant.

### Methods

Three samples, each of 3 mL, were collected from each donor in K_2_EDTA vials (Becton, Dickinson and Company, Gurgaon, India). Three baseline readings were taken from each of the three vials within 30 minutes of collection (thus having 9 baseline readings in total) on the Sysmex XP-100 3-part differential haematology analyser (Sysmex Corporation, Kobe, Japan). The following parameters were studied: WBC, RBC, Hb, Hct, MCV, MCH, MCHC, RDW, Plt and mean platelet volume MPV. The mean of the nine baseline readings of the above parameters were calculated and accepted as the baseline value for each parameter. After the baseline readings, one of the vials was stored at 33 °C.

Samples were stored at 33 °C and analysed after 4, 6, 12, 24, 48 and 72 hours of storage, thus demonstrating the storage stability till or exceeding the time of normal sample retention in the laboratory, which is normally 24 hours on weekdays and 48 hours on weekends. At each time point, three readings of all the parameters from each sample were taken, after which they were re-stored at 33 °C. The mean of the three readings for each parameter at each time point was accepted as the representative value for that parameter at that particular time point.

All the measurements were done on the Sysmex XP-100 in August 2016 with manual feeding of sample by the same person. Quality control of the analyser was maintained throughout the duration of the study by three levels of controls run once a day. Storage temperature was also monitored. The controls were manufactured by Bio-Rad Liquichek™ Hematology controls (Bio Rad Laboratories, Gurgaon, India). For stable controls, at a level corresponding to that in the present study, the coefficient of variations (CV) were 1.1%, 0.7%, 1.1%, 0.6%, 0.3%, 3.9%, 1.6% and 1.3% for WBC, RBC, HGB, HCT, MCV, PLT, RDW and MPV, respectively. Analytical stability was also checked by comparing the values of samples stored at 4 °C overnight to the previous day result.

### Statistical analysis

The percentage difference of each parameter after each storage time compared to the baseline was calculated. The maximum acceptable bias (Bias_max_) was obtained from studies on biological variation (*6*). Median percent difference exceeding the Bias_max_ for a parameter was deemed clinically significant. Statistical significance of the overall variability of each parameter over time was tested by the Friedman test. Statistical significance of the percentage differences was tested by the Wilcoxon signed-rank test using R statistical environment (R Core Team, Vienna, Austria), version 3.1.2, against a null hypothesis of no difference. The P-values were adjusted for multiple comparisons by the Holm method (P_holm_). The bias was deemed unacceptable if there was both clinically significant as well as statistically significant bias at a P_holm_ of 0.05.

If the bias of a parameter at time point was clinically acceptable, the clinical equivalence was tested using two one-tailed Wilcoxon Signed-rank tests (*7*). The percent differences were tested against the null hypothesis that the magnitude of the observed bias was either greater than or equal to the Bias_max_. A parameter at a particular time point was deemed stable if the P_holm_ was less than 0.05.

The 95% confidence interval of the mean percent difference of each parameter after each storage time compared to the baseline was also obtained by studentized pivotal Bootstrap-t (B-t) and bias corrected and accelerated (BCa) bootstrap methods. The 95% confidence interval of the median percent difference was obtained by BCa bootstrap method.

A sample size of 20 showed a power of greater than 99.9% to detect a paired difference of Bias_max_ from baseline value for all parameters other than MCHC at a Bonferroni corrected alpha of 0.05, as estimated by the G*Power program (Universitat Dusseldorf, Dusseldorf, Germany), version 3.1.9.2. For MCHC, the power was approximately 76% at a naive significance level of 0.05.

## Results

Platelets, Hct, MCV, MCHC, RDW and MPV showed unacceptable bias (which was both clinically as well as statistically significant) within 4 hours of storage at 33 °C (Tables 1 and 2). The mean and median of the percent differences and their 95% confidence intervals are given in Supplementary tables 1 and 2.

**Table 1:** Values of parameters tested at different times of storage

**Analytes**	**Baseline**	**4 h**	**6 h**	**12 h**	**24 h**	**48 h**	**72 h**	**P**
**WBC (x10^9^/L)**	8.1 (6.3 - 9.4)	8.1 (6.3 - 9.4)	8.1 (6.2 - 9.3)	7.9 (6.2 -9.2)	7.7 (6.2 - 9)	7.5 (5.9 - 8.7)	7.7 (6.3 - 8.5)	< 0.001
**RBC (x10^12^/L)**	5.1 (4.8 - 5.4)	5.2 (4.9 - 5.4)	5.2 (4.9 - 5.4)	5.2 (4.8 - 5.4)	5.2 (4.8 -5.4)	5.1 (4.8 - 5.3)	5.2 (4.8 - 5.4)	< 0.001
**Hb (g/L)**	151 (143 - 153)	151 (143 - 154)	151 (144 - 154)	152 (143 - 155)	153 (144 - 155)	153 (145 - 156)	153 (146 - 156)	< 0.001
**Hct (L/L)**	0.447 (0.432 – 0.466)	0.453 (0.443 - 0.476)	0.457 (0.442 – 0.475)	0.471 (0.460 - 0.492)	0.499 (0.484 – 0.519)	0.511 (0.494 – 0.542)	0.527 (0.503 – 0.555)	< 0.001
**MCV (fL)**	87.8 (84.4 - 89.4)	89.0 (86.0 - 90.2)	89.7 (86.3 - 91.0)	92.4 (89.3 - 93.8)	97.4 (94.1 - 99.0)	100.7 (97.4 - 103.2)	101.8 (98.3 - 105.0)	< 0.001
**MCH (pg)**	29.3 (28.7 - 30.5)	29.1 (28.2 - 30.6)	29.2 (28.5 - 30.3)	29 (28.4 - 30.6)	29.1 (28.8 - 30.7)	29.6 (29 - 31)	29.9 (29.1 - 31.3)	< 0.001
**MCHC (g/L)**	337 (332 - 341)	331 (322 - 336)	329 (324 - 332)	321 (311 - 324)	304 (298 - 311)	299 (291 - 303)	294 (289 - 302)	< 0.001
**RDW (%)**	14.2 (13.2 - 14.6)	14.5 (13.5 - 14.9)	14.5 (13.6 - 15.1)	15.2 (13.9 - 15.5)	15.7 (14.3 - 16.1)	15.4 (13.9 - 15.8)	15.4 (14.8 - 16.3)	< 0.001
**Plt (x10^9^/L)**	270 (243 - 329)	252 (204 - 296)	239 (207 - 288)	242 (208 - 290)	251 (206 - 282)	248 (211 - 279)	255 (212 -285)	< 0.001
**MPV (fL)**	10.1 (9.3 - 11.2)	10.7 (10.0 -12.0)	10.5 (9.9 - 11.8)	10.4 (9.7 - 11.6)	11 (10.1 - 12.5)	11.1 (10.3 - 12.4)	11.0 (10.1 - 12.1)	< 0.001
WBC - white blood cell. RBC - red blood cell. Hb – haemoglobin. Hct – haematocrit. MCV - mean corpuscular volume. MCH - mean corpuscular haemoglobin. MCHC - mean corpuscular haemoglobin concentration. RDW - red cell distribution width. Plt - platelet count. MPV - mean platelet volume. Data are presented as median (interquartile range).								

**Table 2:** Differences of parameters tested stored at different times from the baseline

**Parameter**	**Biasmax** **(%)**	**Difference from baseline (%)**					
**4 h**	**6 h**	**12 h**	**24 h**	**48 h**	**72 h**
**WBC**	6.0	- 0.06 (- 0.37 - 1.33)	- 0.09 (- 1.13 - 0.85)	- 1.11 (- 2.67 – (- 0.60))	**- 2.69** **(- 3.90 – (- 1.88))**	**- 5.56** **(- 7.78 – (- 4.54))**	**- 5.50** **(- 8.02 – (- 2.80))**
**RBC**	1.7	**0.49** **(0.18 - 0.75)**	**0.54** **(0.32 - 0.75)**	**0.95** **(0.41 - 1.19)**	**0.90** **(0.48 - 1.35)**	0.33 (- 0.49 - 0.75)	0.34 (0.05 - 0.87)
**HGB**	1.8	- 0.04 (- 0.30 - 0.19)	**0.37** **(0.04 - 0.44)**	**0.63** **(0.35 - 0.97)**	**0.94** **(0.69 - 1.18)**	**1.57** **(1.10 - 2.04)**	**2.11** **(1.87 - 2.72)**
**HCT**	1.7	**1.72** **(1.41 - 2.18)**	**2.64** **(2.03 - 3.34)**	**5.97** **(4.94 - 6.91)**	**11.70** **(10.44 - 12.75)**	**15.36** **(13.76 - 16.26)**	**17.62** **(15.04 - 18.45)**
**MCV**	1.2	**1.30** **(1.10 - 1.57)**	2.17 (1.68 - 2.45)	5.28 (4.31 - 5.78)	10.60 (9.99 -11.58)	15.11 (13.49 - 15.72)	16.66 (15.15 - 17.71)
**MCH**	1.3	**- 0.59** **(- 1.09 – (- 0.15))**	- 0.28 (- 0.56 - 0.14)	- 0.31 (- 0.76 – 0.27)	0.22 (- 0.47 - 0.53)	**1.43** **(0.82 - 2.25)**	**2.05** **(1.18 - 2.49)**
**MCHC**	0.4	**- 1.95** **(- 2.33 – (- 1.35))**	- 2.21 (- 2.88 – (- 1.66))	- 5.02 (- 5.64 – (-4.03))	- 9.39 (- 10.43 – (- 8.67))	- 12.28 (-12.68 – (- 10.77))	- 12.53 (- 13.77 - (- 11.36))
**RDW**	1.7	**2.53** **(1.90 - 3.26)**	**3.62** **(3.09 - 4.13)**	**6.66** **(5.91 - 7.83)**	**9.40** **(7.20 - 11.46)**	**8.05** **(4.70 - 11.46)**	**11.39** **(8.03 - 17.78)**
**PLT**	5.9	**- 6.40** **(- 12.93 – (- 5.10))**	**- 7.23** **(- 13.30 – (- 3.97))**	**- 7.77** **(- 12.75 – (- 3.58))**	**- 6.24** **(- 10.55 – (- 2.96))**	**-7.30** **(- 10.80 – (- 2.87))**	**- 4.73** **(- 8.57 – (- 0.83))**
**MPV**	2.2	**6.33** **(5.26 - 8.67)**	**4.51** **(3.88 - 8.05)**	**4.45** **(2.66 - 6.72)**	**8.22** **(3.70 -11.15)**	**9.35** **(7.14 - 12.10)**	**6.35** **(4.13 - 9.68)**
WBC - white blood cell. RBC - red blood cell. Hb – haemoglobin. Hct – haematocrit. MCV - mean corpuscular volume. MCH - mean corpuscular haemoglobin. MCHC - mean corpuscular haemoglobin concentration. RDW - red cell distribution width. Plt - platelet count. MPV - mean platelet volume. Biasmax - maximum acceptable bias. Data are presented as median (interquartile range). P < 0.05 was considered statistically significant. Bolded results are statistically significant.							

**Supplementary Table 1:** Mean and 95% confidence intervals for differences of tested parameters stored at different times

**Parameters**		**4 h**	**6 h**	**12 h**	**24 h**	**48 h**	**72 h**
WBC	**Mean**	0.20	- 0.14	- 1.57	- 2.96	- 6.28	- 5.78
	**B-t 95% CI**	(- 0.59 - 0.88)	(-0.96 - 0.63)	(-2.38 – (- 0.84))	(- 4.05 – (- 2.08))	(- 7.65 – (- 5.16))	(- 8.00 – (- 3.88))
	**BCa 95% CI**	(- 0.51 - 0.85)	(-0.87 - 0.59)	(-2.28 - (- 0.91))	(- 3.99 – (- 2.14))	(- 7.48 - (- 5.25))	(- 7.85 – (- 4.07))
RBC	**Mean**	0.61	0.66	0.8	0.84	0.14	0.38
	**B-t 95% CI**	(0.36 - 0.97)	(0.39 - 1.1)	(0.5 - 1.09)	(0.48 - 1.13)	(- 0.32 - 0.5)	(0.07 - 0.67)
	**BCa 95% CI**	(0.39 - 0.9)	(0.4 - 1.01)	(0.52 - 1.05)	(0.52 - 1.11)	(- 0.26 - 0.47)	(0.09 - 0.65)
HGB	**Mean**	- 0.06	0.31	0.63	0.99	1.6	2.2
	**B-t 95% CI**	(- 0.25 - 0.11)	(0.13 - 0.51)	(0.46 - 0.79)	(0.82 - 1.26)	(1.28 - 1.95)	(1.68 - 2.53)
	**BCa 95% CI**	(- 0.23 - 0.1)	(0.14 - 0.49)	(0.47 - 0.77)	(0.84 - 1.23)	(1.3 - 1.91)	(1.73 - 2.5)
HCT	**Mean**	2.01	2.79	5.95	11.59	14.83	16.22
	**B-t 95% CI**	(1.66 - 2.56)	(2.37 - 3.3)	(5.39 - 6.56)	(10.92 - 12.19)	(13.59 - 15.82)	(12.93 - 17.79)
	**BCa 95% CI**	(1.69 - 2.48)	(2.41 - 3.24)	(5.43 - 6.50)	(10.96 - 12.12)	(13.76 - 15.75)	(13.51 - 17.61)
MCV	**Mean**	1.38	2.11	5.09	10.66	14.67	15.76
	**B-t 95% CI**	(1.22 - 1.6)	(1.91 - 2.33)	(4.61 - 5.56)	(10.12 - 11.16)	(13.78 - 15.44)	(12.31 - 17.26)
	**BCa 95% CI**	(1.23 - 1.5)	(1.92 - 2.31)	(4.66 - 5.52)	(10.18 - 11.11)	(13.88 - 15.40)	(12.97 - 17.04)
MCH	**Mean**	- 0.68	- 0.37	- 0.25	0.08	1.45	1.78
	**B-t 95% CI**	(- 1.1 – (- 0.31))	(- 0.89 - 0.02)	(- 0.60 - 0.06)	(- 0.24 - 0.4)	(1.01 - 1.87)	(1.22 - 2.17)
	**BCa 95% CI**	(- 1.05 – (- 0.35))	(- 0.82 - 0)	(- 0.56 - 0.02)	(-0.21 - 0.38)	(1.03 - 1.83)	(1.27 - 2.13)
MCHC	**Mean**	- 2.00	- 2.38	- 5.00	- 9.51	- 11.49	- 11.96
	**B-t 95% CI**	(- 2.61 – (- 1.57))	(- 3.03 – (- 1.89))	(- 5.65 – (- 4.41))	(- 10 – (- 8.99))	(- 12.27 – (- 10.42))	(- 13.1 – (- 9.78))
	**BCa 95% CI**	(- 2.54 – (- 1.61))	(- 2.93 – (- 1.93))	(- 5.6 – (- 4.49))	(- 9.96 – (- 9.05))	(- 12.19 – (- 10.59))	(- 13.01 – (- 10.22))
PLT	**Mean**	- 9.45	- 10.31	- 10.11	- 8.80	- 8.91	- 6.49
	**B-t 95% CI**	(- 17.47 – (- 6.38))	(- 16.87 – (- 6.44))	(- 15.85 – (- 5.04))	(- 15.57 – (- 5.41))	(- 13.33 – (- 2.63))	(- 2.2 - 0.85)
	**BCa 95% CI**	(- 13.56 – (- 6.66))	(- 15.71 – (- 6.72))	(- 15.63 – (- 6.82))	(- 14.46 – (- 5.42))	(- 13.95 – (- 5.64))	(- 11.94 – (- 2.89))
RDW	**Mean**	2.54	3.63	6.74	9.15	8.65	12.93
	**B-t 95% CI**	(2.09 - 2.94)	(3.02 - 4.16)	(5.98 - 7.64)	(7.72 - 10.63)	(6.45 - 11.41)	(9.91 - 16.79)
	**BCa 95% CI**	(2.15 - 2.91)	(3.09 - 4.12)	(6.05 - 7.55)	(7.8 - 10.48)	(6.66 - 11.05)	(10.18 - 16.35)
MPV	**Mean**	7.04	5.70	4.65	7.92	9.37	6.76
	**B-t 95% CI**	(5.81 - 8.86)	(4.43 - 7.40)	(2.97 - 6.38)	(5.51 - 10.58)	(7.24 - 11.40)	(4.74 - 8.78)
	**BCa 95% CI**	(5.89 - 8.68)	(4.54 - 7.20)	(3.14 - 6.26)	(5.75 - 10.32)	(7.41 - 11.28)	(4.83 - 8.65)
The confidence intervals have been calculated by studentized pivotal Bootstrap-t (B-t) and bias corrected and accelerated (BCa) bootstrap methods. WBC - white blood cell. RBC - red blood cell. Hb – haemoglobin. Hct – haematocrit. MCV - mean corpuscular volume. MCH - mean corpuscular haemoglobin. MCHC - mean corpuscular haemoglobin concentration. RDW - red cell distribution width. Plt - platelet count. MPV - mean platelet volume. CI – confidence interval.							

**Supplementary Table 2:** Median and 95% confidence intervals for percent differences of tested parameters at different storage times

**Parameters**	**4 h**	**6 h**	**12 h**	**24 h**	**48 h**	**72 h**
**WBC**	- 0.06 (- 0.36 - 1.14)	- 0.09 (- 1.1 - 0.78)	-1.11 (- 2.59 - (- 0.76))	- 2.69 (- 3.73 - (-2.15))	- 5.56 (- 7.56 - (-4.70))	- 5.5 (- 8.03 - (- 3.3))
**RBC**	0.49 (0.18 - 0.67)	0.54 (0.33 - 0.72)	0.95 (0.42 - 1.14)	0.9 (0.5 - 1.19)	0.33 (- 0.46 - 0.68)	0.34 (0.09 - 0.67)
**HGB**	- 0.04 (- 0.3 - 0.15)	0.37 (0.07 - 0.41)	0.63 (0.36 - 0.9)	0.94 (0.71 - 1.08)	1.57 (1.13 - 1.99)	2.11 (1.87 - 2.62)
**HCT**	1.72 (1.45 - 2.1)	2.64 (2.04 - 3.19)	5.97 (4.96 - 6.75)	11.7 (10.51 - 12.61)	15.36 (14.33 - 16.13)	17.62 (15.22 - 18.16)
**MCV**	1.3 (1.11 - 1.52)	2.17 (1.74 - 2.43)	5.28 (4.4 - 5.67)	10.6 (10.08 - 11.45)	15.11 (13.58 - 15.69)	16.66 (15.54 - 17.61)
**MCH**	- 0.59 (- 0.99 - (- 0.24))	- 0.28 (- 0.45 - 0.05)	- 0.31 (- 0.76 - 0.15)	0.22 (- 0.43 - 0.49)	1.43 (0.85 - 2.01)	2.05 (1.25 - 2.28)
**MCHC**	- 1.95 (- 2.31 - (- 1.43))	- 2.21 (- 2.77 - (- 1.87))	- 5.02 (- 5.64 - (- 4.26))	- 9.39 (-10.38 - (- 9.02))	- 12.28 (- 12.61 - (-11.31))	- 12.53 (- 13.72 - (- 11.7))
**PLT**	- 6.4 (- 12.76 - (- 5.42))	- 7.23 (- 13.04 - (- 4.53))	- 7.77 (- 11.94 - (- 5.15))	- 6.24 (- 9.93 - (- 3.77))	- 7.3 (- 10.42 - (- 3.42))	- 4.73 (- 8.54 - (- 1.54))
**RDW**	2.53 (1.92 - 3.03)	3.62 (3.13 - 3.93)	6.66 (6.02 - 7.31)	9.40 (7.54 - 10.86)	8.05 (4.86, 10.45)	11.39 (8.18 - 15.99)
**MPV**	6.33 (5.26 - 8.01)	4.51 (3.85 - 6.86)	4.45 (2.34 - 6.09)	8.22 (4.12 - 10.64)	9.35 (7.31, 11.31)	6.35 (4.22 - 9.02)
The 95% confidence intervals have been calculated by bias corrected and accelerated bootstrap (BCa) method. Bootstrap-t was not done due to difficulty in estimating the variance of the median. WBC - white blood cell. RBC - red blood cell. Hb – haemoglobin. Hct – haematocrit. MCV - mean corpuscular volume. MCH - mean corpuscular haemoglobin. MCHC - mean corpuscular haemoglobin concentration. RDW - red cell distribution width. Plt - platelet count. MPV - mean platelet volume.						

When tested for equivalence, the stable parameters were found to be RBC till 72 hours (P_holm_: less than 0.001 at all time-points) and WBC, Hb and MCH till 24 hours (P_holm_ for WBC count and Hb: less than 0.001 up to 24 hours, P_holm_ for MCH: 0.009, 0.002, less than 0.001 and less than 0.001 at 4, 6, 12 and 24 hours respectively). WBC and Hb at 48 hours failed the equivalence tests.

## Discussion

The present study demonstrates the appearance of systematic bias for Hct, MCV, MCHC, RDW, Plt and MPV when stored for 4 hours at 33 ˚C, as well as the stability of WBC, RBC, Hb and MCH results when stored for 24 hours at the same temperature. The rapid appearance of bias at 33 ˚C shows that the present recommendation of sample storage up to 6 or 8 hours is inadequate at high temperatures (*1*, *2*). This study also shows the importance of temperature-controlled storage and transport boxes in blood camps and extra-institutional sample collection centres in areas exposed to high temperatures. In case such precautions cannot be taken (*e.g.* in a resource poor setting), interpretation should be done cautiously. RDW and Hct become unreliable markers. MCH may be preferable to MCV or MCHC. For Plt counts, an initial decrease of about 10% within the first few hours may be taken into account.

Other studies have also found rapid appearance of significant bias in Plt counts on storage above 30 °C (*4*, *8*). Significant shift in RDW and MCHC within 6 hours has also been reported (*4*). In contrast to the present study, clinically significant bias in MCV was not reported after storage above 30 °C for 6 to 8 hours, possibly due to a lower precision in these studies as a result of not taking replicates. Also, a comparison of the present study with some of the previously published studies is not possible due to lack of numerical data for most results, and due to taking a lenient equivalence threshold of 10% (*8*, *9*). Also, equivalence for analytes on storage for ostensibly stable parameters was not analysed in these studies.

In the present study, the samples stored at 33 °C were brought to room temperature at the time of analysis during the duration of the study, and then again restored to 33 °C. Such repeated “heat-cold” cycles may affect the analytical results. However, the primary conclusion of the study (*i.e.* the appearance of unacceptable bias even before 4 hours for most of the parameters after storage at 33 °C), remains unaffected by this limitation since the bias in these cases appeared before any “heat- cold “cycle. The acceptable bias for RBC, Hb, MCH and WBC for at least 24 hours at 33 °C should also be a robust finding; these parameters remain stable despite the potentially destabilizing influence of the “heat-cold” cycles.

The samples were taken from a healthy population; however, differences from patient samples are likely to be mild and should not affect the conclusions of the study. Also, storage stability may depend on the analyser used (*10*). Sysmex XP-100 uses impedance counts; counters using other techniques (*e.g.* optical counts) may show different results.

The present study has tried to fill a gap in published knowledge about the extent of systematic error arising due to sample storage arising in warm ambient temperatures using tests of difference as well as equivalence. In doing so, it demonstrates the stability of Hb, RBC, WBC and MCH for at least 24 hours. More importantly, it demonstrates rapid appearance of bias in RBC volume related parameters (Hct, MCV, RDW, MCHC) as well as Plt and MPV after sample storage at 33 °C, even within the currently published guideline of 6 hours. Thus the present study points out the necessity of special haematological sample storage guidelines for laboratories where such high ambient temperatures are common.
